# How large should the next study be? Predictive power and sample size requirements for replication studies

**DOI:** 10.1002/sim.9406

**Published:** 2022-04-08

**Authors:** Erik W. van Zwet, Steven N. Goodman

**Affiliations:** ^1^ Department of Biomedical Data Sciences Leiden University Medical Center Leiden The Netherlands; ^2^ Departments of Epidemiology and Population Health and of Medicine Stanford University Stanford California USA; ^3^ Stanford Program on Research Rigor and Reproducibility (SPORR) Stanford University School of Medicine Stanford California USA; ^4^ Meta‐research Innovation Center at Stanford (METRICS) USA

**Keywords:** actual power, clinical trial, Cochrane Review, predictive power, type S error

## Abstract

We use information derived from over 40K trials in the Cochrane Collaboration database of systematic reviews (CDSR) to compute the replication probability, or predictive power of an experiment given its observed (two‐sided) P‐value. We find that an exact replication of a marginally significant result with P=.05 has less than 30% chance of again reaching significance. Moreover, the replication of a result with P=.005 still has only 50% chance of significance. We also compute the probability that the direction (sign) of the estimated effect is correct, which is closely related to the type S error of Gelman and Tuerlinckx. We find that if an estimated effect has P=.05, there is a 93% probability that its sign is correct. If P=.005, then that probability is 99%. Finally, we compute the required sample size for a replication study to achieve some specified power conditional on the p‐value of the original study. We find that the replication of a result with P=.05 requires a sample size more than 16 times larger than the original study to achieve 80% power, while P=.005 requires at least 3.5 times larger sample size. These findings confirm that failure to replicate the statistical significance of a trial does not necessarily indicate that the original result was a fluke.

## 1 INTRODUCTION

Independent confirmation of research claims is considered to be an essential element of the scientific method. However, meta‐research has shown that many statistically significant results do not “replicate” in the sense of yielding another significant result when the experiment is repeated exactly, see Reference 1 for the most widely known empirical examples. This realization has given rise to the so‐called replication crisis.^2^, ^3^, ^4^, ^5^, ^6^

It is wrong to interpret a nonsignificant result in a replication experiment as disconfirming or contradicting the original experiment.^7^, ^8^, ^9^, ^10^, ^11^ The Cochrane Collaboration enterprise takes the proper approach of evidence aggregation (meta‐analysis), to interpret the findings of multiple trials of varying significance.^10^ However, prospectively it is often desired to design a replication experiment to have a high chance of supplying moderate to strong evidence against the null hypothesis, that is, statistical significance, if a scientifically important difference exists. This is particularly true in regulatory settings when, as is the case with the U.S. FDA, two statistically significant pivotal trials (or one pivotal and one supportive) are typically needed by the Agency for drug approvals. It is also needed in fields where nonsignificance is improperly interpreted as supporting a null effect, or when such studies are less likely to be published.

The terminology around the concept of statistical power is somewhat confusing, see Reference 12. We will discuss three different kinds of power:

1. Prestudy, or “planned” power: The probability of achieving a statistically significant result if the intervention has a given prespecified effect size, calculated before the experiment is performed.
2. Actual power: The probability of achieving a statistically significant result under the true effect of the intervention. Because we never observe the true effect, the actual power of a particular study is also not observed. However, it is possible to estimate the distribution of the actual power across a (large) collection of studies.
3. Predictive power, or the power of a replication study: Conditional on the results of a given study, it is the probability of a statistically significant effect in a subsequent study of specified sample size (which may differ from that used in the original study.)

We will not discuss “conditional power,” which is used for trial interim monitoring. It is the probability of achieving a statistically significant result if an observed interim effect size is the true effect and the trial continues to its planned end. Finally, there is also “post‐hoc power” which the power of a completed experiment if the observed effect (usually nonsignificant) was the true one. It is not useful both because it is the probability of observing a result after a result has been observed and because it ignores the uncertainty of the estimated effect. Therefore post‐hoc power should be avoided, for example References 13, 14.

The goal of this paper is to provide a method to answer the following questions that arise after an initial study has been conducted:

- What is the probability that a replication study of the same size will yield a significant result in the same direction?
- What is the probability that the estimated effect of a replication study has the same direction as the original estimate?
- What is the probability that the estimated effect of the initial study has the correct direction?
- What is the required sample size for a replication study to achieve some specified power?

We will address these questions by combining the information from the initial study with prior information derived from individual studies in the Cochrane Database of Systematic Reviews (CDSR). The Cochrane collaboration is a global independent network that aims to gather and summarize the best evidence—usually randomized trials—from medical research. While there is evidence that the database may still suffer from some publication bias and dubious research practices such as p‐hacking,^15^ the CDSR is currently the most comprehensive collection of evidence on medical interventions. See Reference 16 for a detailed description of the CDSR.

For simplicity, we will use a three‐number summary of a clinical trial: (β,b,s). Here β is the true effect under investigation. For example, patients treated with a particular drug might have a lower risk of mortality compared to those given a placebo. Such an effect is often expressed as a log hazard ratio, log odds ratio, or risk difference. In other situations, the effect could be the mean difference in a continuous measure in its natural units. A well‐conducted clinical trial yields an unbiased estimate of this effect, b, together with its estimated standard error s. The z‐value equals b/s, making the result of the trial statistically significant at the 5% level (two‐sided), if |z|≥1.96. Lastly, we define the signal‐to‐noise ratio SNR=β/s where we think of β as the “signal” and s as the “noise.”

Most investigators calculate predictive power by specifying a prior probability distribution for the effect β. Here, we will take a different approach and instead determine a prior for the SNR. Since the SNR depends on the standard error s, it is affected by the study design, particularly the sample size. So, while the effect β is in essence a property of nature, the SNR is a combination of nature and design. However, in designed experiments such as clinical trials the two are closely linked through sample size calculations or through the conventions of a field. For a convincing clinical trial, a sufficient number of subjects must be included to be able to estimate interesting or plausible effects with sufficient accuracy. On the other hand, it is unethical (and costly) to burden more subjects than needed. To balance these requirements, a sample size calculation (or interim monitoring) is conducted that has the effect of constraining the SNR. For example, if a trial is to have 80% planned power at (two sided) level α=.05 for an prespecified effect size β, then the sample size must be chosen such that SNR=β/s=2.8. Note that 2.8 is the sum of the 2.5th percentile (1.96) and the 80th percentile (0.84) of the standard normal distribution. In other words, a study with 80% planned power is designed to detect an SNR of 2.8 with 80% probability.

There is a very simple relation between the z‐value and the SNR, so one can estimate the distribution of the SNR from a sample of observed z‐values (Section 2). In fact, we can even estimate the joint distribution of z‐value and the SNR. This joint distribution is not sufficient to make inferences about β without additional assumptions, but it *is* sufficient to make inferences about a number of important statistical properties that depend on (β,b,s) only through z and SNR:

1. exaggeration: |b/β|=|z/SNR|
2. coverage: {b−1.96s<β<b+1.96s}⇔{z−1.96<SNR<z+1.96}
3. significance: {|b|>1.96 s}⇔{|z|>1.96}
4. correct sign: {b·β>0}⇔{z·SNR>0}

In References 17, 18, we focused first two of these (exaggeration and coverage), whereas in this paper, we focus on the last two; the statistical significance and direction (or “sign”) of the observed effect.

This paper is organized as follows. In Section 2, we use the approach from References 17, 18 to estimate the marginal distribution of the SNR across more than 40 000 studies from the CDSR. Since the actual power is just a transformation of the SNR, this immediately gives us the marginal distribution of the actual power among the studies in the CDSR. The estimates we obtain are nearly the same as those reported in^17^, ^18^ which were based on a subset of approximately 20 000 studies that we could positively identify as randomized controlled trials (RCTs). We report them here because both the method and results are relevant for the present paper. In Section 3, we compute the conditional distribution of the actual power given the observed absolute z‐value or, equivalently, the two‐sided P‐value. The mean of this conditional distribution is particularly useful. It can be interpreted as the probability that an exact replication of a randomly selected study from the CDSR with a particular P‐value will yield a significant result in the same direction as the original study. This probability is referred to as the “predictive power” by References 7, 11, 19, 20, 21. However, these authors use a uniform prior on β to compute the predictive power, whereas we construct a prior on the SNR empirically from the z‐values in the CDSR. This makes a substantial difference.

While statistical significance is an important characteristic to consider, there are many alternatives, see References 22, 23, 24. In Section 4, we consider the conditional probability that the direction (or sign) of an estimated effect is correct. This is closely related to the type S error probability of Gelman and Tuerlinckx.^25^, ^26^ We also study Killeen's replication probability $p_{\text{rep}}$ which is the probability that a replication experiment yields an estimated effect in the same direction as the original study.^27^

In Section 5, we turn to sample size calculations for replication studies. In particular, we compute the sample size that is needed such that a replication study has a certain desired probability to achieve significance. We end with a brief discussion.

## 2 ACTUAL POWER ACROSS THE CDSR

We abstract the result of a study as a triple (β,b,s) where β is the parameter or “effect” of interest, such as a difference of means, log odds ratio, or log hazard ratio. We will assume that b is an unbiased, normally distributed estimator of β with standard error s. We will ignore small sample issues by assuming that s is known. Also, we define the z‐value z=b/s and the SNR=β/s. In this paper, we will focus on the joint distribution of the z‐value and the SNR. Note that the z‐value is just the SNR plus independent standard normal noise.

The power of the two‐sided test of $H_{0}:\beta =0$ at level 5% is

(1) $$\begin{array}{ll} P(|b|>1.96\;s|\beta ,s) & =P(|z|>1.96|\text{SNR})\\ & =\Phi (\text{SNR}-1.96)+1-\Phi (\text{SNR}+1.96), \end{array}$$

where Φ is the standard normal cumulative distribution function. The power includes the possibility of a significant result in the wrong direction, which is sometimes called a type III error. In the context of replication, it is more relevant to consider only the probability of obtaining a significant result with the correct sign, which is simply Φ(|SNR|−1.96). We define

(2) pow(x)=Φ(|x|−1.96),

and refer to pow(SNR) as the *actual* power. The actual power should not be confused with the “planned” power that studies are typically designed to have against a particular alternative that is considered to be of clinical or scientific interest.

**FIGURE 1:**
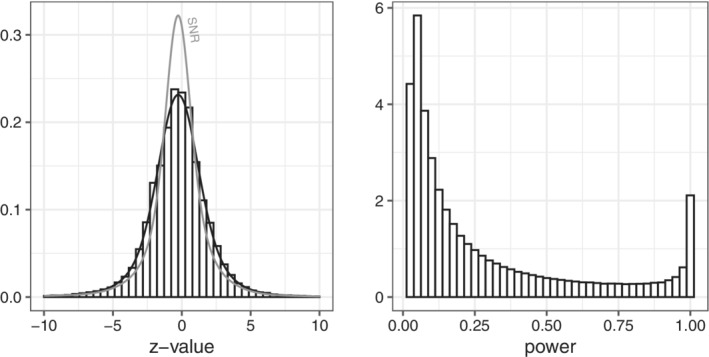
Left panel: The empirical distribution of 45 955 z‐values from the Cochrane Database of Systematic Reviews (CDSR) together with the fitted mixture distribution of four normal components. Also shown (in grey) the distribution of the signal‐to‐noise ratio which was obtained by deconvolution. Right panel: Histogram representing the distribution of the actual power across the CDSR

The Cochrane database is arguably the largest and most reliable collection of evidence in medicine. From this important resource, 45,955 z‐values were derived that represent primary efficacy parameters from unique studies.^28^ We show the histogram of the observed z‐values in Figure 1. It is shifted slightly to the left, which can be explained as follows. The CDSR always compares the experimental condition to the control condition and since the majority of outcomes is binary, a negative z‐value means that the event of interest occurred less often under the experimental condition than under the control condition. Since the event of interest is often unfavorable (death, recurrence of disease, presence of symptoms) a negative z‐value suggests benefit of the experimental treatment. Also, to the extent that the literature is biased toward publication of statistically significant beneficial results, this could manifest as a small nonzero mean of this distribution.

The empirical distribution of the z‐values may appear Gaussian, but in fact it has heavier tails. We decided to model the distribution in a flexible way as mixture of normal distributions. We have used the R package “flexmix,” which implements the EM algorithm, to estimate the parameters. We tried 1 up to 6 components, and found that more than four components makes little difference in the estimated distribution. We added this fitted four‐part mixture distribution to the histogram in Figure 1. The mixture's four components have 3+4+4=11 parameters in total, which are given in Table A1. In References 17, 18, we studied a subset of about 20 000 z‐values where we could positively identify the study as a RCT. The distribution of this subset is virtually identical to the complete set. However, in our previous work we restricted the mixture components to have mean zero, which we did not do here.

Since the z‐value is the sum of the SNR and standard normal noise, its distribution is the *convolution* of the distribution of the SNR and the standard normal density. Hence, we can obtain the distribution of the SNR from the distribution of the z‐value by the process of *deconvolution*, see References 17, 18, 30, 31, 32. Since we are working with a normal mixture, this is very straightforward. We can simply subtract 1 from the variance of each of the mixture components. The resulting standard deviations of the distribution of the SNR are given in the bottom row of Table A1. We also show the estimated density of the SNR in Figure 1.

The actual power, pow(SNR), is just a transformation of the SNR. We generated a sample of size 1 million from the estimated normal mixture distribution of the SNR, and transformed it into a sample from the distribution of the actual power by using (2). We show the histogram in Figure 1. The U‐shape is to be expected on theoretical grounds, see Reference 33. We estimate there is a 12% probability that the actual power is at least 80%. The median of the actual power is 15%, and the mean is 29%. The mean actual power can also be estimated directly by the proportion of significant results in the Cochrane data which is nearly identical at 30%.

It may be surprising to some that the actual power is often so much lower than the planned (or at least desired) power, which is conventionally 80% to 90%. However, the planned power is calculated assuming there is an effect of specified size, defined either explicitly or implicitly. That effect is typically greater than the true effect for most studies in the CDSR. Clearly, finding new treatments with large beneficial effects is difficult. Also, it is well known that with resource and patient limitations, most researchers do not power their studies for the minimum important or likely difference, but instead choose either an overly optimistic difference that reduces sample size requirements, or simply cite the difference with 80% power that corresponds to available resources.

## 3 PREDICTIVE POWER

We have estimated the marginal distribution of the SNR, and it follows from our assumptions that the conditional distribution of z given the SNR is normal with mean SNR and standard deviation 1. Taken together, we actually have the *joint* distribution of z and the SNR. Using standard normal theory (see the Appendix), we can also obtain the conditional distribution of the SNR given z. It is again a normal mixture distribution.

In this paper, we prefer to condition on the absolute z‐value, which is equivalent to conditioning on the two‐sided P‐value. Conditioning on |z| instead of z does represent a (modest) loss of information. However, we believe that it is the “safer” option. While in many trials a negative z‐value might indicate a treatment benefit, this is not the case in every trial. Therefore it is unclear which information is present in the sign.

Since the actual power is just a transformation of the SNR, we also have the conditional distribution of the actual power given |z|. The conditional expectation of the actual power given |z| has an interesting interpretation. It is the conditional probability of a successful replication of a study from the Cochrane database. By “successful replication” we mean obtaining a statistically significant result in the same direction as the original study. This probability is sometimes referred to as the predictive power, for example, Reference 19. We stress that we are *not* claiming that statistical significance is an appropriate method to determine if a replication supports or contradicts the original result. See for example a recent discussion by Held.^11^

**FIGURE 2:**
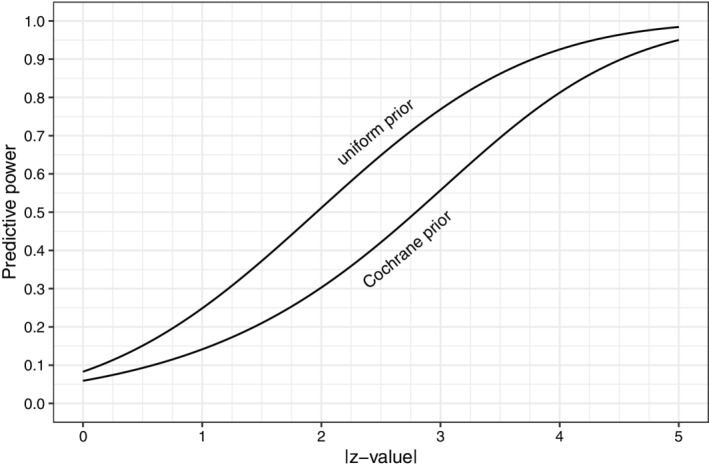
Predictive power based on the uniform prior and the Cochrane prior. We show the post hoc power for comparison only

In Table 1 and Figure 2 we show the predictive power. For example, we see that if a study is —just—significant with P=.05 (ie, |z|=1.96), then the probability that the replication will be significant in the same direction is about 29%.

**TABLE 1:** Columns 1 and 2: The (two‐sided) P‐value and (absolute) z‐value of the original study. Columns 3 and 4: Predictive power, that is, the probability that an exact replication study is significant and the sign of the estimated effect is the same as in the original study. Columns 5 and 6: Probability that the signs of the estimated effect in the original study and the replication study are the same. Columns 7 and 8: Probability that the sign of the estimated effect of the original study is correct. All probabilities are reported assuming either the uniform prior or the prior estimated from the Cochrane data

		Predictive power		Replication of the sign		Original sign correct	
P‐value	z‐value	uniform prior	Cochrane	uniform prior	Cochrane	uniform prior	Cochrane
.5	0.67	0.18	0.11	0.68	0.62	0.75	0.69
.3	1.04	0.26	0.15	0.77	0.68	0.85	0.78
.1	1.64	0.41	0.23	0.88	0.78	0.95	0.90
.05	1.96	0.50	0.29	0.92	0.83	0.98	0.93
.03	2.17	0.56	0.34	0.94	0.86	0.98	0.95
.01	2.58	0.67	0.44	0.97	0.90	1.00	0.98
.005	2.81	0.73	0.50	0.98	0.92	1.00	0.99
.001	3.29	0.83	0.64	0.99	0.96	1.00	1.00

Predictive power, sometimes called the “replication probability," has been proposed before.^7^, ^19^, ^20^, ^21^, ^33^, ^34^ See Kunzmann et al for a very recent review.^35^ However, these authors used the uniform prior for the SNR which means that conditionally on the observed z‐value, the distribution of the SNR is normal with mean z and standard deviation 1. Consequently, the distribution of the z‐value of an exact replication experiment is normal with mean z and standard deviation $\sqrt{2}$. We show the resulting predictive power in Table 1 and Figure 2. We see that, for most z‐values, it is much larger than the predictive power we obtain from using the Cochrane data. This means that traditional approaches are quite overoptimistic about a replication reaching significance. Even for an observed P=.001, the predictive power using the CDSR prior is only 64%. The predictive power does not exceed 80% until the observed z‐value is almost 4.

## 4 THE DIRECTION OF THE EFFECT

Until now, we have focused on the power of the test whether the effect β is zero. However, it has been argued that it is neither plausible nor relevant if β is exactly zero. John Tukey famously put it as follows:^36^

Statisticians classically asked the wrong question – and were willing to answer with a lie, one that was often a downright lie. They asked “Are the effects of A and B different?” and they were willing to answer “no.” All we know about the world teaches us that the effects of A and B are always different – in some decimal place – for any A and B. Thus asking “Are the effects different?” is foolish. What we should be answering first is “Can we tell the direction in which the effects of A differ from the effects of B?”

Our analysis of the primary efficacy outcomes from the CDSR confirms this. Under the estimated distribution of the SNR, the probability is zero that β is exactly zero. Following Tukey's advice, we will now consider the conditional probability that the original estimate has the same sign (direction) as the true effect, given the absolute z‐value, that is,

(3) P(b·β>0||z|)=P(z·SNR>0||z|).

This is closely related to the type S (sign) error probability,^25^, ^26^ which is defined as P(b·β>0||z|>1.96).

**FIGURE 3:**
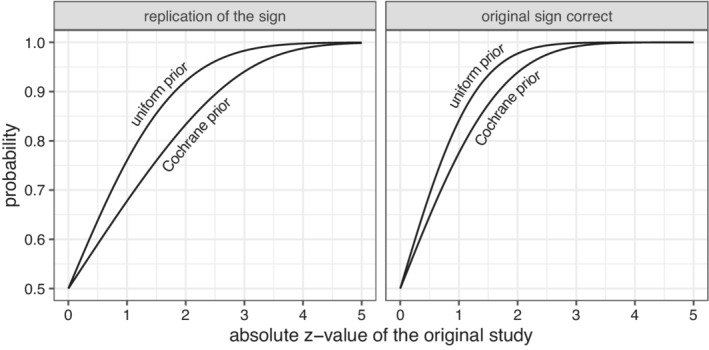
Left panel: Probability that the sign of the original estimate is the same as the replication estimate, given the absolute z‐value of the original trial. Right panel: Probability that the sign of the original estimate is correct, given the absolute z‐value. All probabilities are shown assuming either the uniform prior or the prior estimated from the Cochrane data

If we assume the uniform prior then we have P(z·SNR>0||z|)=Φ(|z|), but we can also compute this probability based on the prior information from the Cochrane data. We show the results in Figure 3 and in Table 1. Again, we note that the probability based on the uniform prior tends to be too optimistic. It is also interesting to note that if z=1.96 then there is a 93% probability that the sign is correct. In other words, the probability that a statistically significant result from the Cochrane database has the correct sign is at least 93%. This is related to the interpretation of the P‐value a measure of the probability on the true effect being in the opposite direction.^37^ Even though 93% is quite high, it is important to note that replication efforts will often not be statistically significant, and so may be misinterpreted as favoring a null effect.

Killeen^27^, ^38^ proposed to consider the probability that the replication estimate has the same sign (direction) as the original estimate. If we denote the estimate and the z‐value of the replication experiment by $b_{r}$ and $z_{r}$, then Killeen's “replication probability” is

$$p_{\text{rep}}=P(b\cdot b_{r}>0||z|)=P(z\cdot z_{r}>0||z|).$$

To compute this probability, he assumes (tacitly in Reference 27, explicitly in Reference 38) that the SNR has the (improper) uniform prior. Then, conditionally on |z|, the SNR has the normal distribution with mean |z| and standard deviation 1 and hence $z_{r}$ has the normal distribution with mean |z| and standard deviation $\sqrt{2}$. So,

$$p_{\text{rep}}=\Phi (|z|/\sqrt{2}).$$

Of course, we can also compute $P(z\cdot z_{r}>0||z|)$ if we assume the prior for the SNR we obtained from the Cochrane data. We show the results in Table 1. We note that the probability based on the uniform prior is typically much larger than the probability based on the Cochrane data. This means that if one would exactly replicate a study from the Cochrane database, the probability of finding an effect in the same direction is smaller than what one would expect from Killeen's $p_{\text{rep}}$.

## 5 SAMPLE SIZE MULTIPLIER

We used the conditional distribution of the SNR given the absolute z‐value to compute the probability of a statistically significant replication study (predictive power). The underlying assumption is that the effect β and the standard error s of the replication study are the same as in the original study. In practice, it is often decided to increase the low actual power of the original study by increasing the sample size. For example, if the replication study is three times larger than the original study, then the standard error will be smaller by a factor $\sqrt{3}$. Hence, the SNR will be larger by a factor $\sqrt{3}$. This is such a simple relation that we can easily compute the predictive power of a replication study with a different sample size. It then follows that we can compute the factor by which we must multiply the sample size of the original study so that the probability of a successful replication is some specific value, such as 80% or 90%. A similar approach was proposed by Micheloud and Held, but using the uniform prior for the SNR.^21^

**FIGURE 4:**
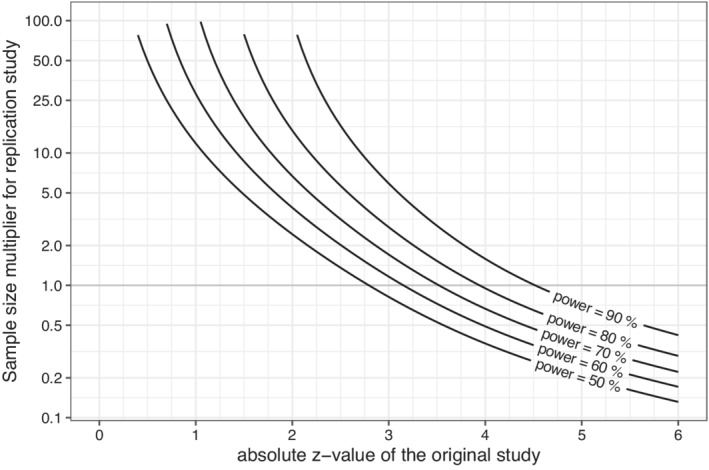
Sample size multiplier that is needed to reach a particular probability of a statistically significant replication (in the same direction), given the absolute z‐value of the original study. Note the logarithmic scale of the y‐axis

In Figure 4 and Table 2 we show the sample size multipliers. For example, we see that if a study is just significant with |z|=1.96, then we need about 2.6 times the sample size so that the probability of successful replication is 50%. We would need about 16 times the sample size to reach 80% probability of successful replication.

**TABLE 2:** Multiplier for the sample size of the original study, to have 50%, 80%, or 90% actual power of the replication study. It is not always possible to reach the required power, which is then indicated by “np”

P‐value	z‐value	Multiplier 50%	Multiplier 80%	Multiplier 90%
.500	0.67	26.8	np	np
.300	1.04	10.9	np	np
.100	1.64	3.9	41.7	np
.050	1.96	2.6	16.3	133.7
.030	2.17	2.0	10.2	45.0
.010	2.58	1.3	5.0	13.4
.005	2.81	1.0	3.6	8.3
.001	3.29	0.6	1.9	3.8

It is not always possible to choose a sample size for a replication study to obtain a desired probability that the replication will be significant in the same direction as the original study. If the SNR has a different sign from the original z‐value, that is, the effect estimate of the original had the wrong sign, then the probability of a successful replication cannot exceed 1/2.

## 6 DISCUSSION

The calculations in this paper demonstrate what many investigators have learned through hard experience, and that numerous meta‐research studies across many fields such as Button et al^4^ and Dumas‐Mallet et al^39^ have found, namely that a large proportion of studies, particularly clinical trials, are not sufficiently powered to provide at least moderate evidence against the null hypothesis (ie, P≤0.05) both when first designed and then repeated, unless the second study is many fold larger than the first. This is true when the first study is statistically significant, and even more so when it is not. This is probably why most meta‐analyses that comprise the CDSR must incorporate many studies, increasing the effective sample size of the evidence base, regardless of statistical significance.

We define the actual power as the probability of obtaining a significant result at the 5% level (two‐sided) under the *true* effect. This should not be confused with the planned power, which is the target of sample size calculations. Typically, the sample size of an experiment is chosen to ensure 80% or 90% probability to detect the “minimal effect you would not want to miss.”^40^ The fact that the actual power is often much lower than the planned power, has various causes: experimental treatments often do not yield the benefit that was hoped for, experiments are powered for overoptimistic effect sizes, or sample sizes are chosen without honest estimates for power for minimally important effects. It is not easy to find new treatments that work well, or to power studies for minimally important effects.

We are able to demonstrate the above mathematically using a novel insight, namely that the distributions of z‐values across a large collection of human subjects studies is informative about the effects measured in all of them. It has been previously thought that priors on the effect sizes themselves were necessary to make valid inferences; these are typically restricted to narrow, disease or therapeutic‐specific domains, an difficult to estimate individually. z‐values are almost universally measured, and agnostic to outcome measure (eg, continuous or binary, multiplicative or additive effect). The difficulty and subjectivity involved in those estimates of priors led most statisticians to use uniform priors to estimate the quantities we have examined. It is taken to represent prior ignorance, which leads researchers to believe that it is a fair and safe choice. In fact, the opposite is true; a very wide prior represents the information that the parameter in question—the SNR in our case—is likely to be very large. However, as we demonstrated empirically, a large SNR, or equivalently high power, is actually quite rare in clinical research, for example References 1, 4.

This confirms that plausible effect sizes and z‐values are linked in the design of human‐subject studies; we calculate sample sizes not just to avoid underpowering but also “overpowering.” Conducting studies on human subjects far larger than they need to be is not just a waste of resources but also ethically problematic. Large studies with ongoing monitoring are typically halted if z‐values are high. So the range and distribution of z‐values across many thousands of human‐subjects interventional and observational studies are informative about what is to be expected in any one study.

This allowed us to calculate actual power, predictive power, and the sample size needed in a single or combination of future experiments to provide moderate to strong evidence (ie P≤.05) after a first experiment, conditional on its result. If the first experiment is statistically significant, and it is desired for a second one to be so as well for regulatory or other purposes, then the sample size of the subsequent experiment must be many‐fold larger than the original to an extent perhaps not previously appreciated. Of course, from an inferential point of view it is not necessary for such evidence to be provided in a single experiment if they are all ultimately to be combined. But that makes many assumptions about how the biomedical research enterprise is funded, how many future trials will be motivated or justified, and how evidence is interpreted.

Since we estimate the joint distribution of the z‐value and the SNR from all trials in the CDSR, our results refer to what would happen if we chose a trial from the CDSR (or from the population of all trials that are “exchangeable” with the CDSR) completely at random and we observe a particular P‐value (or absolute z‐value). For example, if we select an original study with P=0.05, then it has a 93% probability of the correct sign (Table 3). The probability of an exact replication reaching statistical significance is 29% (Table 2). In our opinion, these calculations suggest we reconsider common teaching and intuitions about the replication of studies.

The question of whether a particular trial is “exchangeable” with the trials in the CDSR deserves attention. In a 2011 review, Davey et al examined approximately 112,000 CDSR trials.^16^ The median sample size was 91 (IQR 44‐210). Highest sample sizes were shown for cancer, infectious diseases, and pregnancy and childbirth, with small sample sizes in studies of mental health and behavioral conditions. However, different sample sizes do not necessarily connote different z‐value distributions. Pharmacologic therapy was involved in a large majority of interventions, and the median number of studies per meta‐analysis was only 3. For any particular trial there may be specific information that justifies using a prior that differs from that derived from the entire CDSR. However, such a information would have to be based on high‐quality evidence to be generally agreed on. Therefore, we believe that in the absence of such information, the general information from the CDSR provides an appropriate frame of reference for the interpretation or planning of biomedical human subjects studies.

Finally, we want to comment briefly on the relation of the present paper to a number of other studies. In References 17, 18, 32 we estimated the joint distribution of the z‐values and the SNRs of the primary efficacy outcomes in the CDSR. Since the actual power (ie, the power against the true effect) is a transformation of the absolute value of the SNR, we can derive both the marginal distribution and the conditional distribution given |z| of the actual power. We refer to the conditional mean of the actual power given |z| as the predictive power, and this is the main focus of the present paper.

Turner et al^41^ used all the studies from the CDSR with a binary outcome to estimate the power against a *particular* effect, namely a 30% relative risk reduction. This is a different a different objective from ours, but Turner et al. did reach a similar qualitative conclusion that most studies in the CDSR have low power.

A recent paper by Stanley et al^42^ focuses on the actual power (which they refer to as the retrospective power). They estimate the actual power of a number of studies from meta‐analyses of comparable studies. We treated all studies in the CDSR separately, and did not use the fact that the CDSR is actually a collection of meta‐analyses. Thus, Stanley et al are mainly focused on the interpretation and reliability of meta‐analyses whereas we are mainly concerned with interpreting single studies.

Stanley et al also assumed that there are null effects and nonnull effects, and focus on the familiar 2×2 table of false and true positives and negatives. We treated the effect measure as continuous. We found that under the estimated distribution of the SNR, the probability of a true null effect among the primary efficacy outcomes of the trials in the CDSR is zero. Therefore, instead of deciding whether the effect is null or nonnull, we studied whether it has the correct sign.^25^, ^26^

## Supporting information

Appendix S1. Supporting information

## Data Availability

We provide a supplemental R Markdown document which reproduces all the Figures and Tables of this paper from a publicly available data source (Appendix S1).^28^

## DISTRIBUTION OF THE SNR

### 1

We have estimated the distribution of the z‐value as a mixture of four normal components. We obtain the distribution of the SNR by deconvolution, which boils down to subtracting 1 from the variance of each component. Thus, the two distributions are as shown in Table A1.

**TABLE A1:** Mixture distributions of the z‐value and the signal‐to‐noise ratio (SNR) estimated from 45 955 z‐values from the Cochrane Database of Systematic Reviews (CDSR)

	comp.1	comp.2	comp.3	comp.4
Proportions	0.33	0.31	0.30	0.06
Means	−0.28	−0.22	−0.25	−1.05
SD of the z‐value	1.27	1.60	2.57	5.94
SD of the SNR	0.78	1.25	2.37	5.85

Suppose the SNR is distributed as a mixture of k normal components with mixture proportions $p_{1},\ldots ,p_{k}$, means $\mu_{1},\ldots ,\mu_{k}$ and standard deviations $\sigma_{1},\ldots ,\sigma_{k}$. In our case, k=4. Then z is also a mixture of k normals with the same mixture proportions and means, but standard deviations $\sqrt{\sigma_{i}^{2}+1}$. The density of z is

(A1)
$$g(z)=\overset{k}{\underset{i=1}{\sum }}\frac{p_{i}}{\sqrt{\sigma_{i}^{2}+1}}\;\varphi \left(\frac{z-\mu_{i}}{\sqrt{\sigma_{i}^{2}+1}}\right),$$

where φ is the standard normal density function.

The conditional distribution of the SNR given z is again a mixture of normals. The mixing proportions are

(A2)
$$q_{i}=\frac{p_{i}}{\sqrt{\sigma_{i}^{2}+1}}\varphi \left(\frac{z-\mu_{i}}{\sqrt{\sigma_{i}^{2}+1}}\right)\frac{1}{g(z)},$$

the means of the components are

(A3)
$$m_{i}=\frac{\mu_{i}+z\;\sigma_{i}^{2}}{\sigma_{i}^{2}+1},$$

and the variances of the components are

(A4)
$$v_{i}^{2}=\frac{\sigma_{i}^{2}}{\sigma_{i}^{2}+1}.$$
