# Supplementary material for: How large should the next study be? Predictive power and sample size requirements for replication studies
Source: Stat Med. 2022 Apr 8;41(16):3090–101. doi: 10.1002/sim.9406 (PMC9325423; doi:10.1002/sim.9406)
Supplement: Supplementary file 1 — Appendix S1. Supporting information [file SIM-41-3090-s001.pdf]

Supplement: How large should the next study be?

Erik van Zwet

07 maart, 2022

Contents

1 **Packages and utility functions** 1

2 **Introduction** 2

3 **Data** 3

4 **Distribution of z-value and SNR** 4

5 **Actual power** 5

6 **Predictive power** 6

7 **Probability of the same sign** 9

8 **Sample size multiplier** 11

1 Packages and utility functions

```
library(dplyr)
library(tidyr)       # for gather()

library(ggplot2)     # for plotting
library(cowplot)
library(geomtextpath)

library(kableExtra)  # for tables
library(xtable)

library(flexmix)     # for fitting normal mixture


dmix = function(x,p,m,s){    # density of normal mixture (vector x)
  p %*% sapply(x, function(x) dnorm(x,mean=m,sd=s))
}
```

```
pmix = function(x,p,m,s){     # cdf of normal mixture (vector x)
  drop(p %*% sapply(x, function(x) pnorm(x,mean=m,sd=s)))
}

rmix = function(n,p,m,s){     # density of normal mixture
  d=rmultinom(n,1,p)
  rnorm(n,m%*%d,s%*%d)
}


powerfun = function(z){
  crit=qnorm(0.975)
  # 1-pnormabs(1.96,m=abs(z),1)    # pnorm(-1.96-z) + 1 - pnorm(1.96-z)
  # pnorm(abs(z)-crit)             # significant two-sided test in same direction
  1-pnorm(crit,abs(z),1)
}

posterior <- function(z,p,m,s) {   # p(SNR | z) when snr ~ dmix(p,m,s)
  s2 <- s^2
  p <- p*dnorm(z,m,sqrt(s2+1))
  p <- p/sum(p)                    # conditional mixing probs
  m <- z*s2/(s2+1) + m/(s2+1)      # conditional means
  v <- s2/(s2+1)                   # conditional variances
  s <- sqrt(v)                     # conditional std devs
  data.frame(p,m,v,s)
}
```

2 Introduction

Consider the triple (β, b, s) where

- β is the parameter of interest
- b is an unbiased, normally distributed estimator of β
- s is the standard error of b.

Also, define the z-value $z = b/s$ and the signal-to-noise ratio $SNR = \beta/s$. The power of the two-sided test of $H_0 : \beta = 0$ at level 5% is

$$P(|z| > 1.96|\beta, s) = \Phi(SNR - 1.96) + 1 - \Phi(SNR + 1.96).$$

This probability includes the possibility of a significant result in the wrong direction, which is sometimes called a type III error. In the context of replication, it is more relevant to consider the probability of a significant result *with the same sign* as β is $\Phi(|SNR| - 1.96)$. For this reason, we define the power function

$$\text{pow}(x) = \Phi(|x| - 1.96).$$

We start by plotting this power function.

```
x=seq(-5,5,0.1)
qplot(x,powerfun(x),ylab="Power",geom="path")+
  scale_y_continuous(breaks=seq(0,1,0.1),minor_breaks=seq(0,1,0.05))+
  theme_bw()
```

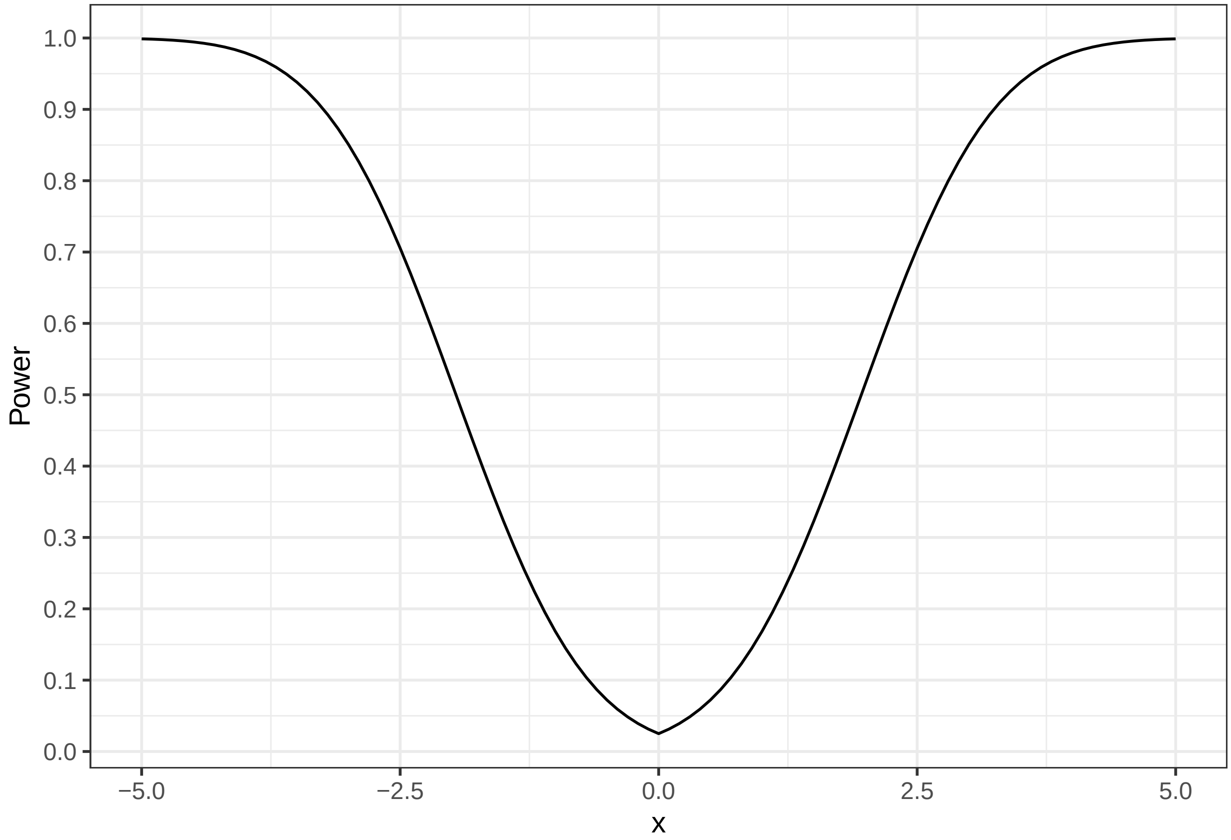

3 Data

We use z-values for the primary efficacy outcome of more than 40,000 trials from the Cochrane database.

```
set.seed(123)  # for reproducibility

data=read.csv("https://osf.io/xq4b2/?action=download")
#data=read.csv("C:/Users/ewvanzwet/OneDrive - LUMC/papers/Cochrane data/CochraneEffects.csv")

d=data[data$outcome.group=="efficacy" & data$outcome.nr==1 & abs(data$z)<20,]
d=group_by(d,study.id.sha1) %>% sample_n(size=1)   # select single outcome per study
z=d$z
cat("number of z-values:",length(z),"\n")
```

```
number of z-values: 45955
```

```
cat("proportion significant:",mean(abs(z)>1.96),"\n")
```

```
proportion significant: 0.2996627
```

4 Distribution of z-value and SNR

We used the data from the Cochrane database to estimate the the marginal distribution of z as a mixture of four normal components.

Next, we can get the distribution of the SNR by deconvolution with the standard normal distribution. That is, we simply subtract 1 from the variances of the normal components of the mixture distribution of z.

```
k=4                                   # number of mixture components
if (TRUE){
  fit=flexmix(z ~ 1, k = k)            # estimate distribution of z=b/s
  p=summary(fit)@comptab$prior         # mixture proportions
  m=parameters(fit)[1,]
  sigma=parameters(fit)[2,]
  ind=order(sigma)
  p=p[ind]                             # mixing proportions of z
  m=m[ind]                             # mixing means of z
  sigma=sigma[ind]                     # standard deviations of z
  tau=sqrt(sigma^2-1)                  # standard deviations of SNR=beta/s
  rbind(p,m,sigma,tau)
}else{
  # distr. of SNR
  p=c(0.33, 0.31, 0.30, 0.06)
  m=c(-0.28,-0.22,-0.25,-1.05)
  sigma=c(1.27, 1.60, 2.57, 5.94)
  tau=c(0.78, 1.25, 2.37, 5.85)
}
```

```
         Comp.3     Comp.1     Comp.2      Comp.4
p      0.3273549  0.3107035  0.3001238  0.06181792
m     -0.2789022 -0.2202724 -0.2469372 -1.04831280
sigma  1.2670937  1.5969190  2.5688924  5.93838618
tau    0.7781558  1.2450503  2.3662646  5.85358270
```

```
distr=rbind(p,m,sigma,tau)
rownames(distr)=c("proportions","means","std. dev. of the $z$-value","std. dev. of the SNR")
colnames(distr)=paste("comp.",1:k,sep='')
distr=round(distr,2)
kable(distr)
```

	comp.1	comp.2	comp.3	comp.4
proportions	0.33	0.31	0.30	0.06
means	-0.28	-0.22	-0.25	-1.05
std. dev. of the z-value	1.27	1.60	2.57	5.94
std. dev. of the SNR	0.78	1.25	2.37	5.85

```
xtable(distr)
```

```
% latex table generated in R 4.0.5 by xtable 1.8-4 package
% Mon Mar 07 23:45:12 2022
\begin{table}[ht]
\centering
\begin{tabular}{rrrrr}
```

```
  \hline
 & comp.1 & comp.2 & comp.3 & comp.4 \\
  \hline
proportions & 0.33 & 0.31 & 0.30 & 0.06 \\
  means & -0.28 & -0.22 & -0.25 & -1.05 \\
  std. dev. of the \$z\$-value & 1.27 & 1.60 & 2.57 & 5.94 \\
  std. dev. of the SNR & 0.78 & 1.25 & 2.37 & 5.85 \\
   \hline
\end{tabular}
\end{table}
```

```
x=seq(-10,10,0.01)
n=length(z)
d1=data.frame(x,y=drop(dmix(x,p=p,m,s=sigma)))
d2=data.frame(x,y=drop(dmix(x,p=p,m,s=tau)))

ggp1=ggplot(d, aes(x=z, y=..density..)) +
  geom_histogram(bins=40,fill="white",col="black") +
  xlim(-10, 10) + theme_bw() + labs(x="z-value",y="") +
  geom_line(data=d1,aes(x=x,y=y)) +
  geom_textline(data=d2,aes(x=x,y=y,label="SNR"),
              hjust=0.52,vjust=-0.5,size=2.5,color="grey50")
```

5 Actual power

Since the power is a function of the SNR, we can easily transform our estimate of the marginal distribution of the SNR into the marginal distribution of the power. We show the histogram of a million samples.

```
snr=rmix(10^6,p,m,tau)
power=powerfun(snr)
df=data.frame(power=power)
ggp2=ggplot(df, aes(x=power, y=..density..)) +
  geom_histogram(bins=40,fill="white",col="black") +
  theme_bw() + labs(x="power",y="")

plot_grid(ggp1, ggp2)
```

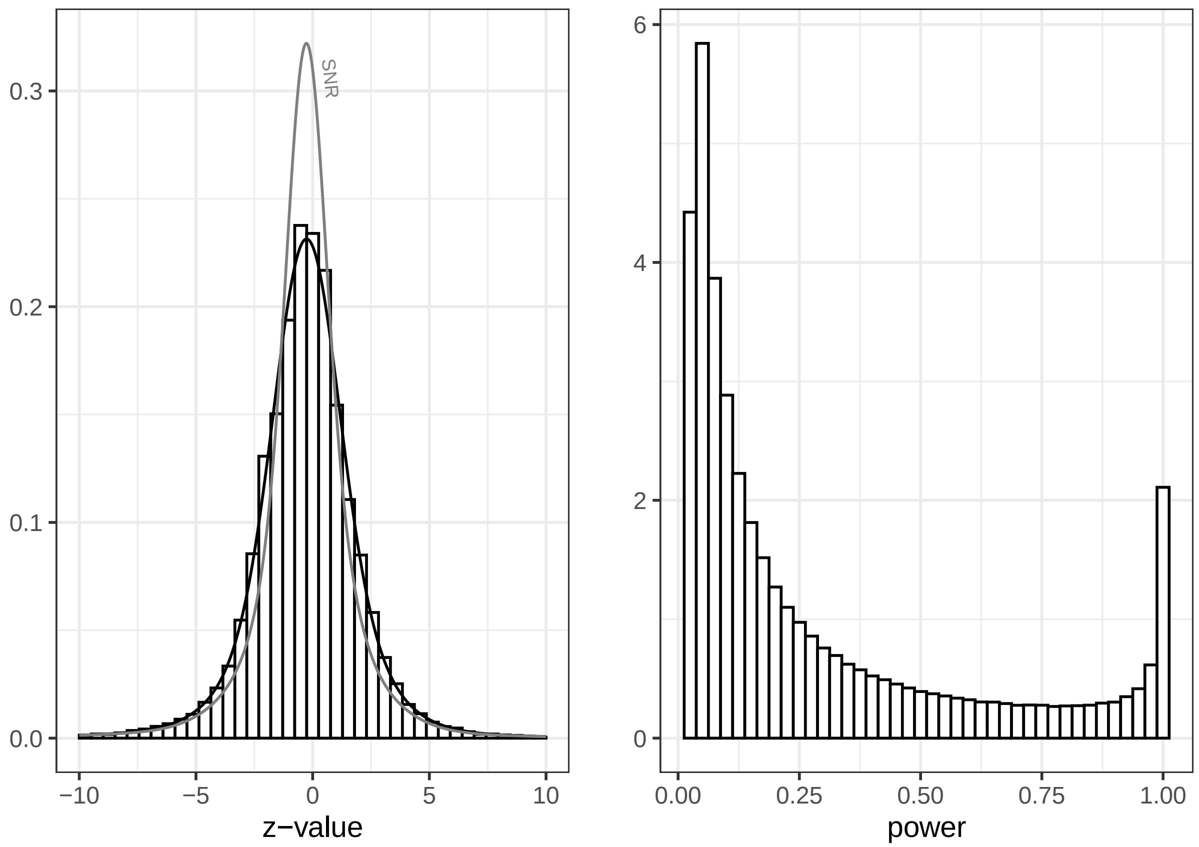


```
ggsave("figures/estimate_z_snr_power.pdf",width=6,height=3)
summary(power)
```

```
##    Min. 1st Qu.  Median    Mean 3rd Qu.    Max.
## 0.02500 0.06104 0.14734 0.28746 0.41496 1.00000
```

6 Predictive power

So, we have a very accurate estimate of the marginal distribution of the SNR, and we know that the conditional distribution of z given the SNR is normal with mean SNR and standard deviation 1. So, we have access to the *joint* distribution of z and the SNR.

The conditional expectation of pow(SNR) given $|z|$ has an interesting interpretation. It is the probability of getting a significant result in the same direction as the original result when the original study is replicated exactly.

The conditional distribution of the SNR given the z-value is a mixture of normals. To condition on $|z|$ we use the fact that for $z > 0$ we have

$$p(SNR \mid |Z| = z) = p(SNR \mid Z = -z)p(Z < 0||Z| = z) + p(SNR \mid Z = z)p(Z > 0||Z| = z)$$

```
pval=c(0.5,0.3,0.1,0.05,0.03,0.01,0.005,0.001)
Z=qnorm(1-pval/2)
Z=c(Z,seq(0,6,0.05))
```

```
#q=seq(0.1,0.9,0.1)


df1=data.frame(Z=Z,pr=NA,target="Cochrane prior", hjust=0.5,vjust=2)

i=0
for (z in Z){
  i=i+1
  z=abs(z)
  pr=dmix(z,p,m,sigma) / (dmix(z,p,m,sigma) + dmix(-z,p,m,sigma))    # pr(z >0 | |z|)
  pr=drop(pr)
  postpos=posterior( z,p,m,tau)          # p(SNR|z= |z|)
  postneg=posterior(-z,p,m,tau)          # p(SNR|z=-|z|)
  powpos=1 - pmix(1.96,p=postpos$p,m=postpos$m,s=sqrt(postpos$v+1))  # signif given z=|z|
  powneg=   pmix(-1.96,p=postneg$p,m=postneg$m,s=sqrt(postneg$v+1))  # signif given z=-|z|
  df1$pr[i]=pr*powpos + (1-pr)*powneg                              # signif given |z|
}
```

We plot the predictive power based on the Cochrane prior together with the predictive power based on the uniform prior. To compute the predictive power based on the flat prior, we have by symmetry for any $z > 0$

$$\begin{aligned} p(SNR \mid |Z| = z) &= p(SNR \mid Z = -z)p(Z < 0||Z| = z) + p(SNR \mid Z = z)p(Z > 0||Z| = z) \\ &= \frac{1}{2}p(SNR \mid Z = -z) + \frac{1}{2}p(SNR \mid Z = z) \\ &= p(SNR \mid Z = z) \end{aligned}$$

```
df2=data.frame(Z=Z,pr=1-pnorm(1.96,Z,sqrt(2)),target="uniform prior",
               hjust=0.55,vjust=-1)

tab=data.frame(zval=Z,predicted_flat=df2$pr,predicted_Cochrane=df1$pr)
tab=cbind(pval,round(tab[1:8,],2))
kable(tab)
```

pval	zval	predicted_flat	predicted_Cochrane
0.500	0.67	0.18	0.11
0.300	1.04	0.26	0.15
0.100	1.64	0.41	0.23
0.050	1.96	0.50	0.29
0.030	2.17	0.56	0.34
0.010	2.58	0.67	0.44
0.005	2.81	0.73	0.50
0.001	3.29	0.83	0.64

```
print(xtable(tab,digits=c(1,3,2,2,2)), include.rownames=FALSE)
```

```
% latex table generated in R 4.0.5 by xtable 1.8-4 package
% Mon Mar 07 23:45:17 2022
\begin{table}[ht]
\centering
\begin{tabular}{rrrr}
  \hline
pval & zval & predicted\_flat & predicted\_Cochrane \\
```

```
  \hline
0.500 & 0.67 & 0.18 & 0.11 \\
  0.300 & 1.04 & 0.26 & 0.15 \\
  0.100 & 1.64 & 0.41 & 0.23 \\
  0.050 & 1.96 & 0.50 & 0.29 \\
  0.030 & 2.17 & 0.56 & 0.34 \\
  0.010 & 2.58 & 0.67 & 0.44 \\
  0.005 & 2.81 & 0.73 & 0.50 \\
  0.001 & 3.29 & 0.83 & 0.64 \\
   \hline
\end{tabular}
\end{table}
```

```
df=rbind(df2,df1)
ggplot(df, aes(x=Z,y=pr,group=target)) +
  geom_textline(aes(label = target,hjust=hjust,vjust=vjust),size=3.5) +
  scale_y_continuous(minor_breaks = seq(0,1,0.05),
                     breaks = seq(0,1,0.1),lim=c(0,1)) +
  scale_x_continuous(minor_breaks = seq(0,5,0.25),
                     breaks = seq(0,5,1),lim=c(0,5)) +
  ylab('Predictive power') + xlab("|z-value|") + theme_bw()
```

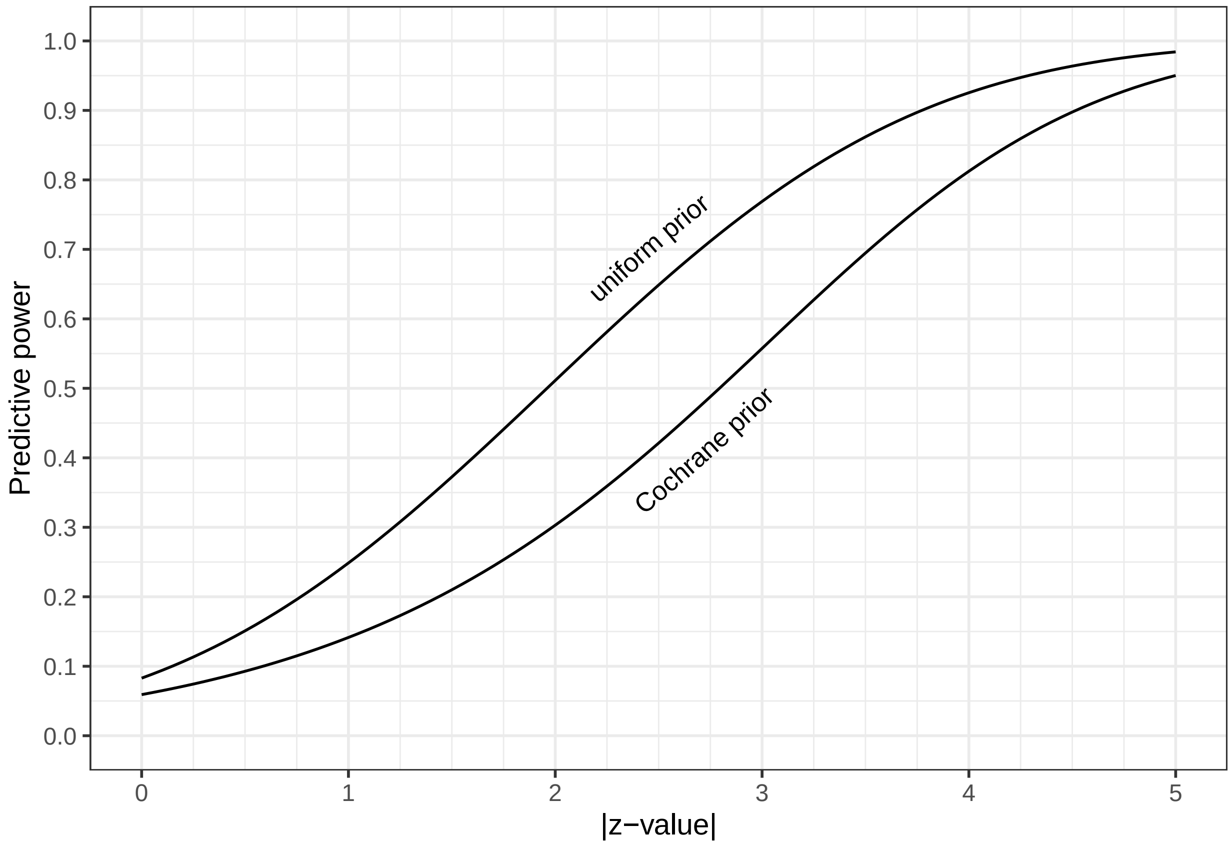


```
ggsave("figures/pred_power.pdf",width=6,height=4)
```

7 Probability of the same sign

```
df3=data.frame(Z=Z,pr=pnorm(abs(Z)/sqrt(2)),target="replication of the sign",
               prior="uniform prior",hjust=0.5,vjust=-0.5)
df4=data.frame(Z=Z,pr=0,target="replication of the sign",
               prior="Cochrane prior",hjust=0.5,vjust=1.5)
df5=data.frame(Z=Z,pr=pnorm(abs(Z)),target="original sign correct",
               prior="uniform prior",hjust=0.5,vjust=-0.5)
df6=data.frame(Z=Z,pr=0,target="original sign correct",
               prior="Cochrane prior",hjust=0.5,vjust=1.5)

k=length(Z)
for (i in 1:k){
  z=abs(Z[i])
  pr=dmix(z,p,m,sigma) / (dmix(z,p,m,sigma) + dmix(-z,p,m,sigma)) # pr(z >0 | |z|)
  pr=drop(pr)

  post=posterior( z,p,m,tau)          # p(SNR|z= |z|)
  pm=post$m
  ps=post$s
  prob1=1 - pmix(0,p=post$p,m=pm,s=sqrt(ps^2 + 1))  # z2 positive given z=|z|
  prob2=1 - pmix(0,p=post$p,m=pm,s=ps)              # SNR positive given z=|z|

  post=posterior(-z,p,m,tau)          # p(SNR|z=-|z|)
  pm=post$m
  ps=post$s
  prob3=pmix(0,p=post$p,m=pm,s=sqrt(ps^2 + 1))  # z2 negative given z=-|z|
  prob4=pmix(0,p=post$p,m=pm,s=ps)              # SNR negative given z=-|z|

  df4$pr[i]=pr*prob1 + (1-pr)*prob3
  df6$pr[i]=pr*prob2 + (1-pr)*prob4
}

tab=data.frame(zval=Z,
               signif_repl_flat=df2$pr,
               signif_repl_Cochrane=df1$pr,
               sign_repl_flat=df3$pr,
               sign_repl_Cochrane=df4$pr,
               sign_truth_flat=df5$pr,
               sign_truth_Cochrane=df6$pr)
tab=cbind(pval,round(tab[1:8,],2))
kable(tab) %>% row_spec(0, angle = -45)
```

pval	zval	signif_repl_flat	signif_repl_Cochrane	sign_repl_flat	sign_repl_Cochrane	sign_truth_flat	sign_truth_Cochrane
0.500	0.67	0.18	0.11	0.68	0.62	0.75	0.69
0.300	1.04	0.26	0.15	0.77	0.68	0.85	0.78
0.100	1.64	0.41	0.23	0.88	0.78	0.95	0.90
0.050	1.96	0.50	0.29	0.92	0.83	0.98	0.93
0.030	2.17	0.56	0.34	0.94	0.86	0.98	0.95
0.010	2.58	0.67	0.44	0.97	0.90	1.00	0.98
0.005	2.81	0.73	0.50	0.98	0.92	1.00	0.99
0.001	3.29	0.83	0.64	0.99	0.96	1.00	1.00

```r
print(xtable(tab,digits=c(1,3,rep(2,7))), include.rownames=FALSE)
```

```
% latex table generated in R 4.0.5 by xtable 1.8-4 package
% Mon Mar 07 23:45:18 2022
\begin{table}[ht]
\centering
\begin{tabular}{rrrrrrrr}
  \hline
pval & zval & signif\_repl\_flat & signif\_repl\_Cochrane & sign\_repl\_flat & sign\_repl\_Cochrane & si
  \hline
0.500 & 0.67 & 0.18 & 0.11 & 0.68 & 0.62 & 0.75 & 0.69 \\
  0.300 & 1.04 & 0.26 & 0.15 & 0.77 & 0.68 & 0.85 & 0.78 \\
  0.100 & 1.64 & 0.41 & 0.23 & 0.88 & 0.78 & 0.95 & 0.90 \\
  0.050 & 1.96 & 0.50 & 0.29 & 0.92 & 0.83 & 0.98 & 0.93 \\
  0.030 & 2.17 & 0.56 & 0.34 & 0.94 & 0.86 & 0.98 & 0.95 \\
  0.010 & 2.58 & 0.67 & 0.44 & 0.97 & 0.90 & 1.00 & 0.98 \\
  0.005 & 2.81 & 0.73 & 0.50 & 0.98 & 0.92 & 1.00 & 0.99 \\
  0.001 & 3.29 & 0.83 & 0.64 & 0.99 & 0.96 & 1.00 & 1.00 \\
   \hline
\end{tabular}
\end{table}
```

```r
df=rbind(df3,df4,df5,df6)
df$target=factor(df$target)
df$target=relevel(df$target,ref="replication of the sign")

ggplot(df,aes(x=Z, y=pr, group=prior)) +
  geom_textline(aes(label=prior,vjust=vjust),size=3,hjust=0.5) +
  scale_y_continuous(minor_breaks = seq(0.5,1,0.05),
                     breaks = seq(0.5,1,0.1),lim=c(0.5,1)) +
  scale_x_continuous(minor_breaks = seq(0,5,0.5),
                     breaks = seq(0,5,1),lim=c(0,5)) +
  xlab("absolute z-value of the original study") +
  ylab("probability") +
  facet_grid(. ~ target) + theme_bw()
```

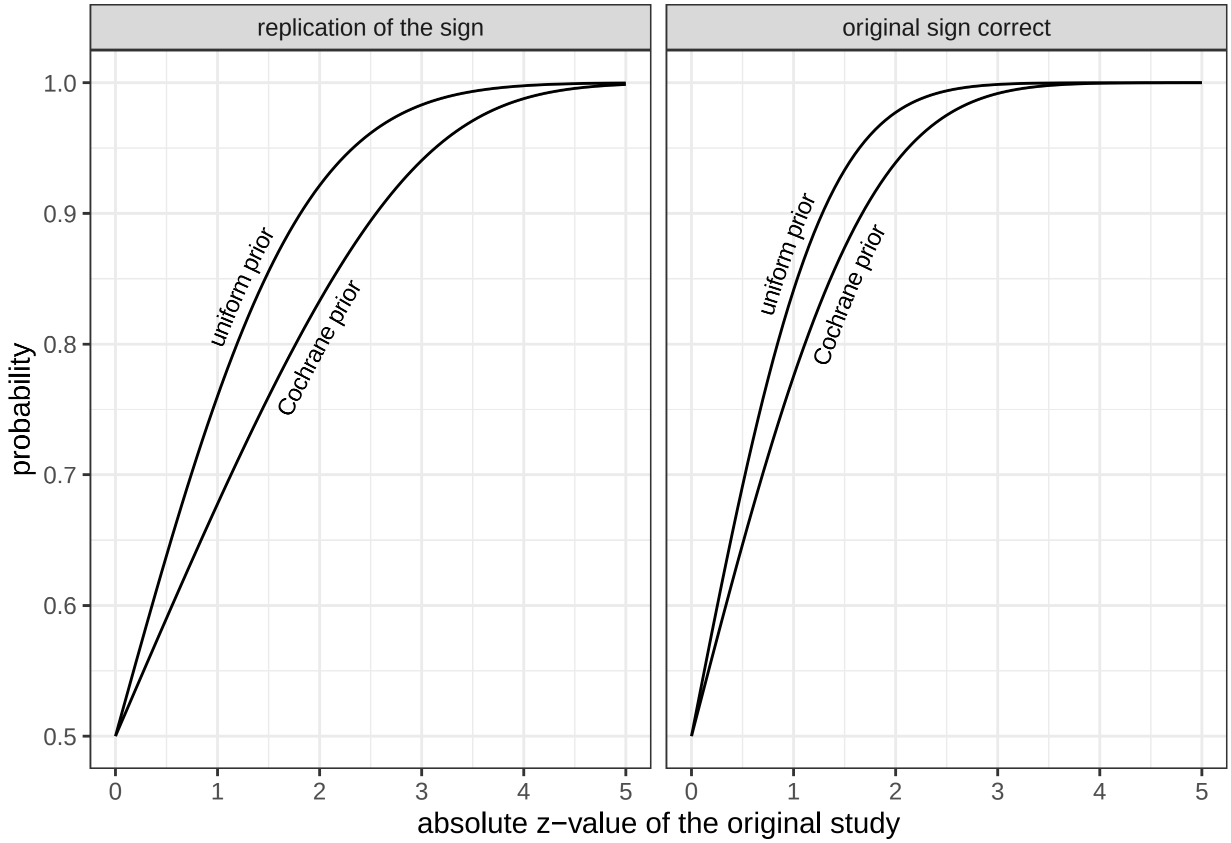


```
ggsave("figures/sign.pdf",width=6,height=3)
```

8 Sample size multiplier

With the Cochrane data we can estimate the prior distribution of the SNR in a typical RCT. When we observe some z-value, we can compute the posterior (conditional) distribution of the SNR. This posterior becomes the prior when we repeat the RCT. Using this prior, we can compute the probability that the second (replication) RCT will be significant, i.e. the predictive power. This is what we did in the previous section.

We can also compute the predictive power when we use a different sample size for the replication experiment. For example, if we use a sample size that is twice as large, the SNR will be larger by a factor of $\sqrt{2}$.

But then we can also turn it around, and compute the sample size for the replication such that the probability of getting a significant result is 80% or 90%.

```
Z=seq(0,6,0.05)
pows=seq(0.5,0.9,0.1)
df=data.frame(Z=NA,pow=NA,multiplier=rep(1,length(Z)*length(pows)))

predpow=function(z,mult){  # compute predictive power when original experiment
                           # produced z, and we multiple the sample size for
                           # the replication
  z=abs(z)
  pr=dmix(z,p,m,sigma) / (dmix(z,p,m,sigma) + dmix(-z,p,m,sigma)) # pr(z >0 | |z|)
  pr=drop(pr)
```

```
  post=posterior( z,p,m,tau)          # p(SNR|z= |z|)
  pm=sqrt(mult)*post$m
  ps=sqrt(mult)*post$s
  powpos=1 - pmix(1.96,p=post$p,m=pm,s=sqrt(ps^2 + 1))  # signif given z=|z|

  post=posterior(-z,p,m,tau)          # p(SNR|z=-|z|)
  pm=sqrt(mult)*post$m
  ps=sqrt(mult)*post$s
  powneg=   pmix(-1.96,p=post$p,m=pm,s=sqrt(ps^2 + 1))  # signif given z=-|z|
  pr*powpos + (1-pr)*powneg                           # signif given |z|
}


multiplier=function(z,power){  # find sample size multiplier to get particular power
                               # after observing z
  z=abs(z)
  if (predpow(z,0.01)<power & predpow(z,1000)>power){
    multiplier=uniroot(function(mult){predpow(z,mult)-power},
                       interval=c(0.01,1000))$root
  }
  else {multiplier=NA}
  return(multiplier)
}

i=0
for (z in Z){
  for (pow in pows){
    i=i+1
    df$Z[i]=z
    df$multiplier[i]=multiplier(z,pow)
    df$pow[i]=pow
  }
}

df$label=paste("power =",100*df$pow,"%")
ggplot(df,aes(x=Z, y=multiplier, group=pow)) +
  geom_hline(yintercept=1,color="grey") +
  geom_textline(aes(label=label),hjust=0.9,size=3) +
  xlab("absolute z-value of the original study") +
  ylab("Sample size multiplier for replication study") +
  scale_x_continuous(breaks=seq(0,6,1),minor_breaks=seq(0,6,0.25))+
  scale_y_continuous(limits=c(NA,100),breaks=c(0.1,0.2,0.5,1,2,5,10,25,50,100),
                     trans="log10") + theme_bw()
```

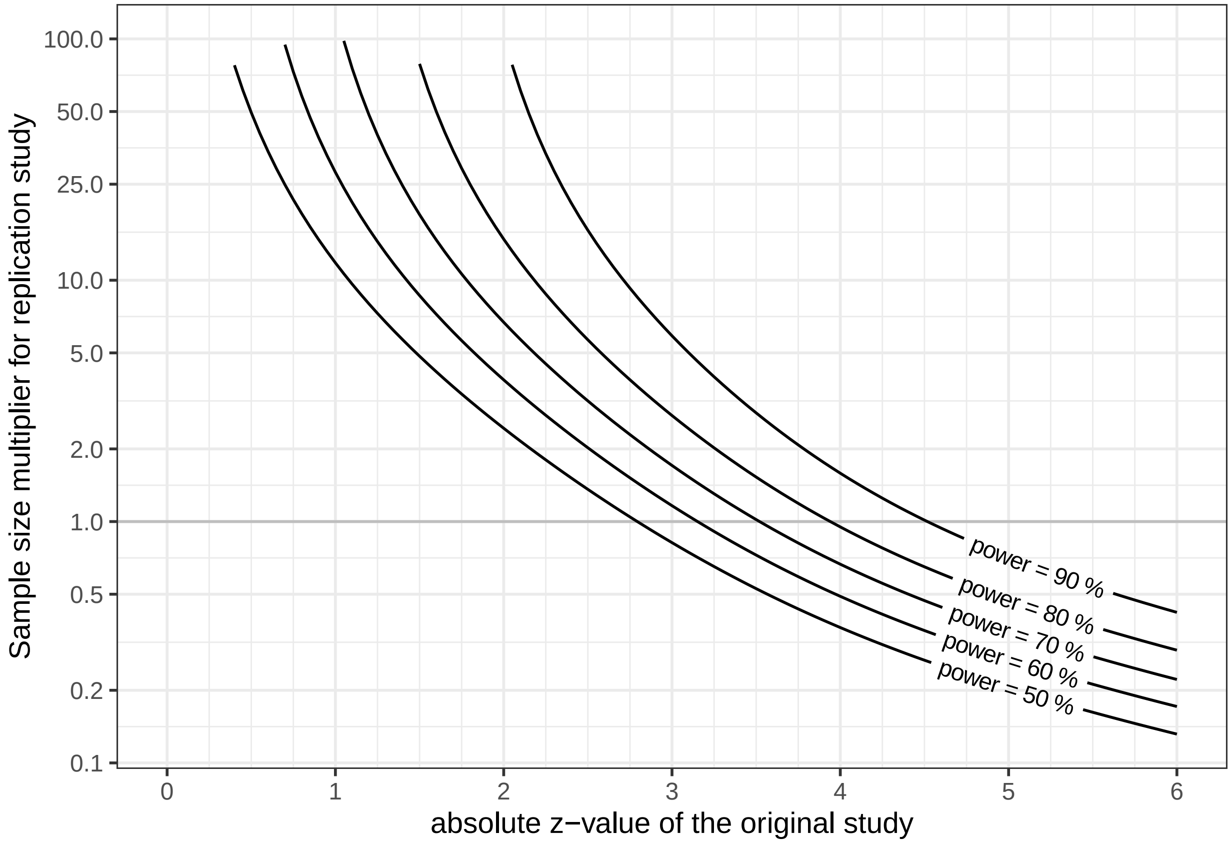


```
ggsave("figures/multiplier.pdf",width=6,height=4)

pval=c(0.5,0.3,0.1,0.05,0.03,0.01,0.005,0.001) # from Goodman, 1992
Z=qnorm(1-pval/2)
tab2=data.frame(pval=pval,zval=Z,multiplier50=NA,
                multiplier80=NA,multiplier90=NA)
i=0
for (z in Z){
   i=i+1
   tab2$multiplier50[i]=multiplier(z,pow=0.5)
   tab2$multiplier80[i]=multiplier(z,pow=0.8)
   tab2$multiplier90[i]=multiplier(z,pow=0.9)
}
ind=order(-tab2$pval)
tab2=tab2[ind,]
kable(tab2)
```

pval	zval	multiplier50	multiplier80	multiplier90
0.500	0.6744898	26.8512269	NA	NA
0.300	1.0364334	10.9384001	NA	NA
0.100	1.6448536	3.9117777	41.818833	NA
0.050	1.9599640	2.5616866	16.284934	134.208590
0.030	2.1700904	1.9788575	10.243616	45.054335
0.010	2.5758293	1.2532008	5.019520	13.435942
0.005	2.8070338	0.9869257	3.567384	8.298322
0.001	3.2905267	0.6284388	1.927663	3.752834

```r
print(xtable(tab2,digits=c(1,3,2,1,1,1)), include.rownames=FALSE)
```

```
% latex table generated in R 4.0.5 by xtable 1.8-4 package
% Mon Mar 07 23:45:38 2022
\begin{table}[ht]
\centering
\begin{tabular}{rrrrr}
  \hline
pval & zval & multiplier50 & multiplier80 & multiplier90 \\
  \hline
0.500 & 0.67 & 26.9 &  &  \\
  0.300 & 1.04 & 10.9 &  &  \\
  0.100 & 1.64 & 3.9 & 41.8 &  \\
  0.050 & 1.96 & 2.6 & 16.3 & 134.2 \\
  0.030 & 2.17 & 2.0 & 10.2 & 45.1 \\
  0.010 & 2.58 & 1.3 & 5.0 & 13.4 \\
  0.005 & 2.81 & 1.0 & 3.6 & 8.3 \\
  0.001 & 3.29 & 0.6 & 1.9 & 3.8 \\
   \hline
\end{tabular}
\end{table}
```
